# Characterizing an Uncertainty Diagram and Kirkwood–Dirac Nonclassicality Based on Discrete Fourier Transform

**DOI:** 10.3390/e25071075

**Published:** 2023-07-17

**Authors:** Ying-Hui Yang, Bing-Bing Zhang, Xiao-Li Wang, Shi-Jiao Geng, Pei-Ying Chen

**Affiliations:** School of Mathematics and Information Science, Henan Polytechnic University, Jiaozuo 454000, China; yangyinghui4149@163.com (Y.-H.Y.); zhangbingbing0803@163.com (B.-B.Z.); gengshijiao@hpu.edu.cn (S.-J.G.); pychen@hpu.edu.cn (P.-Y.C.)

**Keywords:** Kirkwood–Dirac nonclassicality, uncertainty diagram, discrete Fourier transform, complete incompatible bases

## Abstract

In this paper, we investigate an uncertainty diagram and Kirkwood–Dirac (KD) nonclassicality based on discrete Fourier transform (DFT) in a *d*-dimensional system. We first consider the uncertainty diagram of the DFT matrix, which is a transition matrix from basis A to basis B. Here, the bases A, B are not necessarily completely incompatible. We show that for the uncertainty diagram of the DFT matrix, there is no “hole” in the region of the $\left(n_{A},n_{B}\right)$ plane above and on the line $n_{A}+n_{B}=d+1$. Then, we present where the holes are in the region strictly below the line and above the hyperbola $n_{A}n_{B}=d$. Finally, we provide an alternative proof of the conjecture about KD nonclassicality based on DFT.

## 1. Introduction

In quantum mechanics, there exist many nonclassical properties, such as entanglement, discord, coherence, nonlocality, contextuality and negativity or nonreality of quasiprobability distributions. By studying these nonclassical properties, one can not only obtain a better understanding of quantum mechanics but also explore their applications in quantum information processing. The Kirkwood–Dirac (KD) distribution is a quasiprobability distribution that was independently developed by Kirkwood [1] and Dirac [2]. It is a finite-dimensional analog of the well-known Wigner distribution [3,4]. A quasiprobability distribution behaves like a probability distribution, but negative or nonreal values are allowed to appear in the distribution. For a quantum state and some observables, the KD distribution of this state can be obtained. A quantum state is called KD classical if the KD distribution of the state is real non-negative everywhere, i.e., a probability distribution. Otherwise, it is called KD nonclassical. Recently, KD nonclassicality has come to the forefront due to its application in quantum tomography [5,6,7,8,9,10,11,12,13,14] and weak measurements [15,16,17].

The noncommutativity of observables cannot guarantee the nonclassicality of a state [18]. The KD nonclassicality of a state depends not only on the state but also on the eigenbases of observables. Given a state |ψ〉 and an eigenbasis A of observable *A* and an eigenbasis B of observable *B*, authors in Ref. [19] gave a sufficient condition on the KD nonclassicality of a state; that is, |ψ〉 is KD is nonclassical if $n_{A}\left(\psi \right)+n_{B}\left(\psi \right)>\left\lfloor \frac{3d}{2}\right\rfloor$, where $n_{A}\left(\psi \right)$ counts the number of nonvanishing coefficients in the basis A representation, and it is similar for $n_{B}\left(\psi \right)$. In 2021, De Bièvre [20] introduced the concept of complete incompatibility on the eigenbases A,B of two observables A,B and presented the relations among complete incompatibility, support uncertainty and KD nonclassicality, also showing that |ψ〉 is KD nonclassical if $n_{A}\left(\psi \right)+n_{B}\left(\psi \right)>d+1$ and $\langle a_{i}|b_{j}\rangle \neq 0$, where $|a_{i}\rangle$ and $|b_{j}\rangle$ are the eigenvectors of A,B, respectively. Xu [21] generalized the concept of complete incompatibility to *s*-order incompatibility and established a link between *s*-order incompatibility and the minimal support uncertainty. Fiorentino et al. [22] generalized Tao’s uncertainty relation [23] to complete sets of mutually unbiased bases in spaces of prime dimensions. Recently, De Bièvre [24] provided an in-depth study of the links of complete incompatibility to support uncertainty and to KD nonclassicality. Xu [25] gave some characterizations for the general structure of KD classical pure states and showed that when A,B are mutually unbiased bases, |ψ〉 is KD classical if and only if $n_{A}\left(\psi \right)n_{B}\left(\psi \right)=d$. This answered the conjecture in Ref. [20]. Langrenez et al. characterized how the full convex set of states with positive KD distributions depends on the eigenbases of A and B [26].

Discrete Fourier transform (DFT) is an important linear transform in quantum information theory. The uncertainty diagram is a practical and visual tool to study the uncertainty of a state with respect to bases A,B. De Bièvre [24] characterized the uncertainty diagram of complete incompatibility bases. However, for the DFT matrix with nonprime order, the bases A,B are not completely incompatible bases. The uncertainty diagram of the DFT matrix with nonprime order is still unclear. In this paper, we consider this question. Firstly, for the uncertainty diagram of DFT, we show that for any dimension *d*, there is no “hole” in the region of the $\left(n_{A},n_{B}\right)$ plane above or on the line defined by Tao’s uncertainty relation [23], $n_{A}+n_{B}=d+1$, i.e., there is no absence of states with $\left(n_{A}\left(\psi \right),n_{B}\left(\psi \right)\right)$ in the region. Secondly, we provide some holes in the region strictly below the line and above the hyperbola defined by Donoho and Stark’s product uncertainty relation [27], $n_{A}n_{B}=d$. Finally, we give a new method to prove the conjecture [20] about KD nonclassicality of a state based on DFT. Our method avoids analyzing the phases and only uses the concept of congruence class.

The rest of this paper is organized as follows. In Section 2, we recall some relevant notions and notations. In Section 3, we study the uncertainty diagram of DFT. In Section 4, we give an alternative method to characterize the KD nonclassicality of a state based on DFT. Conclusions are given in Section 5.

## 2. Preliminaries

Consider a Hilbert space H with dimension *d*. Let an orthonormal basis $A=\{|a_{i}\rangle {\}}_{i=0}^{d-1}$, respectively, $B=\{|b_{j}\rangle {\}}_{j=0}^{d-1}$, be the eigenbasis of observable *A*, respectively, of observable *B*. Let *U* be the unitary transition matrix with entries $U_{ij}=\langle a_{i}|b_{j}\rangle$ from basis A to basis B. In terms of these two bases, the Kirkwood–Dirac (KD) distribution of a state |ψ〉∈H can be written as

(1) $$Q_{ij}=\langle a_{i}|\psi \rangle \langle \psi |b_{j}\rangle \langle b_{j}|a_{i}\rangle ,i,j\in Z_{d}.$$

It is a quasiprobability distribution and satisfies $\sum_{i,j=0}^{d-1}Q_{ij}=1$, with conditional probabilities $Q\left(a_{i}|\psi \right)=\sum_{j=0}^{d-1}Q_{ij}={\left|\langle a_{i}|\psi \rangle \right|}^{2}$ and $Q\left(b_{j}|\psi \right)=\sum_{i=0}^{d-1}Q_{ij}={\left|\langle b_{j}|\psi \rangle \right|}^{2}$. A state |ψ〉 is called classical if the KD distribution of |ψ〉 is a probability distribution, i.e., $Q_{ij}\geq 0$ for all $i,j\in Z_{d}$. Otherwise, |ψ〉 is called nonclassical. Obviously, all of the basis vectors $|a_{i}\rangle$ and $|b_{j}\rangle$ are classical.

Given a state |ψ〉∈H, let $n_{A}\left(\psi \right)$, respectively, $n_{B}\left(\psi \right)$, be the number of nonzero components of |ψ〉 on A, respectively, on B. That is, $n_{A}\left(\psi \right)=\left|S_{\psi }\right|$ and $n_{B}\left(\psi \right)=\left|T_{\psi }\right|$, where

(2) $$\begin{array}{ccc} S_{\psi } & = & \{i|\langle a_{i}|\psi \rangle \neq 0,i\in Z_{d}\},\\ T_{\psi } & = & \{j|\langle b_{j}|\psi \rangle \neq 0,j\in Z_{d}\}, \end{array}$$

and |·| denotes the cardinality of a set.

Two bases A and B are called completely incompatible [20] if all index sets $S,T\in Z_{d}$ for which |S|+|T|≤d have the property that

(3) $$H\left(S,T\right):=\Pi_{A}\left(S\right)H⋂\Pi_{B}\left(T\right)H=\left\{0\right\},$$

where $\Pi_{A}\left(S\right)$ is an orthogonal projector $\sum_{i\in S}|a_{i}\rangle \langle a_{i}|$ and $\Pi_{A}\left(S\right)H$ is a |S|-dimensional subspace. Notice that any $|\psi \rangle \in \Pi_{A}\left(S\right)H$ implies $S_{\psi }\subseteq S$. If A and B are completely incompatible and mutually unbiased (or close to mutually unbiased), the only classical states are the basis states [24].

The physical interpretation of completely incompatible bases is based on the theory of selective projective measurements [24]. Suppose, on a state |ψ〉, successive measurements in basis A then basis B yields the outcomes *S* then *T*. If the outcome *S* occurs with probability one when measuring again in A after having obtained *T*, then it implies that the measurement B is not disturbed by the first outcome *S*. The completely incompatible bases implies that such repeated compatible selective measurements cannot occur.

The uncertainty diagram for orthonormal bases A,B, denoted by UNCD(A,B), is a set of points $\left(n_{A},n_{B}\right)\in Z_{d+1}^{*}\times Z_{d+1}^{*}$ in the $n_{A}n_{B}$ plane, for which there exists a state |ψ〉 such that $n_{A}\left(\psi \right)=n_{A}$ and $n_{B}\left(\psi \right)=n_{B}$, where $Z_{d+1}^{*}=Z_{d+1}\backslash \left\{0\right\}$. For any state |ψ〉∈H, we have $n_{A}\left(\psi \right)n_{B}\left(\psi \right)\geq$ max${}_{i,j}{\left|\langle a_{i}|b_{j}\rangle \right|}^{-2}$, where $i,j\in Z_{d}$ [20,24,27]. If A,B are mutually unbiased bases (MUBs) [28,29], i.e., $|\langle a_{i}|b_{j}\rangle |=\frac{1}{\sqrt{d}}$ for all $i,j\in Z_{d}$, we have $n_{A}\left(\psi \right)n_{B}\left(\psi \right)\geq d$. This is Donoho and Stark’s product uncertainty relation [27]. This means that all the points $(n_{A},n_{B})\in$ UNCD(A,B) are above or on the hyperbola $n_{A}n_{B}=d$. The inequality $n_{A}+n_{B}\geq d+1$ is Tao’s uncertainty relation [23].

The following lemma was introduced in Ref. [24]. It can be employed to determine whether a point $\left(n_{A},n_{B}\right)$ belongs to UNCD(A,B).

**Lemma** **1.** *Let S,T be two subsets of $Z_{d}$ and suppose dim H(S,T)=L≥1. Suppose that for all $S^{\prime }\subseteq S$ for which $|S^{\prime }|=|S|-1$, one has dim $H(S^{\prime },T)\leq L-1$, and that for all $T^{\prime }\subseteq T$ for which $|T^{\prime }|=|T|-1$, one has dim $H(S,T^{\prime })\leq L-1$. Then, the set of |ψ〉∈H(S,T) for which $n_{A}\left(\psi \right)=\left|S\right|$, $n_{B}\left(\psi \right)=\left|T\right|$ is an open and dense set in H(S,T). The opposite implication is also true.*

By Lemma 1, the point (d,d) belongs to the UNCD(A,B), since dimH(S,T)=dimH=d and $\dim H\left(S^{\prime },T\right)=\dim H\left(S,T^{\prime }\right)=d-1$. In order to better employ Lemma 1, let us first consider subspace H(S,T). Without loss of generality, let S={0,1,…,k−1}, T={0,1,…,l−1}. If k=d, then $H\left(S,T\right)=\Pi_{B}\left(T\right)H$. If k≠d, H(S,T) is isomorphic to the null space of a (d−k)×l matrix. Notice that [20]

(4) $$\begin{array}{cc} H(S,T)≅ & \{\left(\beta_{0},\ldots ,\beta_{l-1}\right)|\langle a_{i}|\varphi \rangle =0,i\in Z_{d}\backslash S\},\\ = & \{\left(\beta_{0},\ldots ,\beta_{l-1}\right)|\sum_{j=0}^{l-1}\langle a_{i}|b_{j}\rangle \beta_{j}=0,i\in Z_{d}\backslash S\}, \end{array}$$

where $|\varphi \rangle =\sum_{j=0}^{l-1}\beta_{j}|b_{j}\rangle \in H\left(S,T\right)$, and ≅ denotes two sets are isomorphic. It follows that H(S,T) is isomorphic to the null space of the matrix $M={\left(\langle a_{i}|b_{j}\rangle \right)}_{(d-k)\times l}$. It implies that dimH(S,T)=l − Rank (M). In this paper, a submatrix of a matrix *U* is denoted by

(5) $$\begin{array}{c} U\left(\begin{array}{c} i_{0},i_{1},\ldots ,i_{s};\\ j_{0},j_{1},\ldots ,j_{t}. \end{array}\right), \end{array}$$

where $i_{k}$ and $j_{l}$ are the $i_{k}$-th row and $j_{l}$-th column of *U* and $i_{k},j_{l}\in Z_{d}$.

Now, an improved lemma is given to show the existence of point $\left(n_{A},n_{B}\right)$ in UNCD(A,B).

**Lemma** **2.** *In UNCD(A,B), suppose $n_{A}\neq d$. Then, a point $(n_{A},n_{B})\in$ UNCD(A,B) if and only if there exists a $\left(d-n_{A}\right)\times n_{B}$ submatrix M of the transition matrix U,*

(6) $$\begin{array}{c} M=U\left(\begin{array}{c} i_{0},i_{1},\ldots ,i_{d-n_{A}-1};\\ j_{0},j_{1},\ldots ,j_{n_{B}-1}. \end{array}\right), \end{array}$$

*which satisfies the following three conditions:*

*(i) Rank$\left(M\right)<n_{B}$;*

*(ii) Rank$(M^{\prime })=$ Rank(M)+1, where*

(7) $$\begin{array}{c} M^{\prime }=U\left(\begin{array}{c} i_{0},i_{1},\ldots ,i_{d-n_{A}-1},i_{d-n_{A}};\\ j_{0},j_{1},\ldots ,j_{n_{B}-1}. \end{array}\right) \end{array}$$

*and $i_{d-n_{A}}\in Z_{d}\backslash \left\{i_{0},i_{1},\ldots ,i_{d-n_{A}-1}\right\}$;*

*(iii) Rank$(M^{\prime \prime })=$ Rank(M), where $M^{\prime \prime }$ is a new submatrix of U that is obtained by removing one column of M.*

**Proof.**  Let us first consider the sufficiency. Without loss of generality, suppose that a submatrix $M={\left(\langle a_{i}|b_{j}\rangle \right)}_{\left(d-n_{A}\right)\times n_{B}}$ with $n_{A}\leq i\leq d-1$ and $0\leq j\leq n_{B}-1$ satisfies the three conditions in Lemma 2. Let $S=Z_{n_{A}}$ and $T=Z_{n_{B}}$.

Note that dimH(S,T) is equal to the dimension of the null space of *M*, that is, $\dim H\left(S,T\right)=n_{B}-$ Rank(M). Then, dimH(S,T)≥1, since Rank$\left(M\right)<n_{B}$. For any $S^{\prime }\subset S$ for which $|S^{\prime }|=n_{A}-1$, assume $S^{\prime }=S\backslash \left\{k\right\},k\in Z_{n_{A}}$. Then, $\dim H(S^{\prime },T)$=$n_{B}-$ Rank$\left(M^{\prime }\right)$, where $M^{\prime }$ is a $\left(d-n_{A}+1\right)\times n_{B}$ submatrix of *U* that is obtained by adding row *k*, i.e.,

$$\begin{array}{c} (\langle a_{k}|b_{0}\rangle ,...,\langle a_{k}|b_{n_{B}-1}\rangle ), \end{array}$$

to *M*. By condition (ii), we have Rank$(M^{\prime })=$ Rank(M)+1. Thus,

$$\begin{array}{ccc} \dim H(S^{\prime },T) & = & n_{B}-\mathrm{Rank}\left(M^{\prime }\right)\\ & = & n_{B}-\mathrm{Rank}\left(M\right)-1\\ & < & \dim H(S,T)). \end{array}$$

For any $T^{\prime }\subset T$ for which $|T^{\prime }|=n_{B}-1$, assume $T^{\prime }=T\backslash \left\{l\right\},l\in Z_{n_{B}}$. Then, $\dim H(S,T^{\prime })$ is equal to the dimension of the null space of $M^{\prime \prime }$, i.e., $\dim H\left(S,T^{\prime }\right)=n_{B}-1-$ Rank$\left(M^{\prime \prime }\right)$, where $M^{\prime \prime }$ is a $\left(d-n_{A}\right)\times \left(n_{B}-1\right)$ submatrix of *U* that is obtained by removing column *l* of *M*, i.e., ${\left(\langle a_{1}|b_{l}\rangle ,\ldots ,\langle a_{n_{A}-1}|b_{l}\rangle \right)}^{T}$. By condition (iii), we have Rank$(M^{\prime \prime })=\;$ Rank(M). It follows $\dim H\left(S,T^{\prime }\right)=n_{B}-1-$ Rank$\left(M^{\prime \prime }\right)=n_{B}-1-$ Rank(M)<dimH(S,T). Therefore, $(n_{A},n_{B})\in$ UNCD(A,B) by Lemma 1.

Now, we turn to showing the necessity. We proceed by contradiction. If condition (i) does not hold, i.e., Rank$\left(M\right)=n_{B}$, it implies dimH(S,T)=0. Thus, $(n_{A},n_{B})\notin$ UNCD(A,B) by Lemma 1. It is a contradiction. If condition (ii) cannot be satisfied, i.e., for any $\left(d-n_{A}\right)\times n_{B}$ submatrix *M* of *U*, there exists a row, called row *k*, that is added to *M* such that Rank$(M^{\prime })=\;$ Rank(M). Hence, $\dim H\left(S^{\prime },T\right)=\dim H\left(S,T\right)=n_{B}-$ Rank(M), where $S^{\prime }=S\backslash \left\{k\right\}$. It means $(n_{A},n_{B})\notin$ UNCD(A,B) by Lemma 1. Similarly, we can also obtain the desired result when condition (iii) cannot be satisfied. □

Note that $M^{\prime }$ in Equation (7) is a submatrix of *U*, and condition (ii) means the rank will increase by one if a new row is added to the submatrix *M*. And condition (iii) means the rank is invariant if a column of *M* is removed. Lemma 2 provides a more efficient method to determine whether a point $\left(n_{A},n_{B}\right)$ belongs to UNCD(A,B) or not.

Now, we introduce the discrete Fourier transform (DFT). Suppose that *F* is the DFT matrix with $F=\left(F_{ij}\right)=\left(\frac{1}{\sqrt{d}}\omega_{d}^{ij}\right)$, where $i,j\in Z_{d}$ and $\omega_{d}=e^{2\pi \sqrt{-1}/d}$. Obviously, *F* is a symmetric and reversible Vandermonde matrix. The DFT matrix *F* has the following property.

**Lemma** **3.** *Suppose m|d but m≠d. Let*

(8) $$\begin{array}{cc} M= & F\left(\begin{array}{c} i_{0},i_{0}+m,\cdots ,i_{0}+\left(t-1\right)m;\\ j_{0},j_{1},\cdots ,j_{s-1}. \end{array}\right), \end{array}$$

*where $t\leq \frac{d}{m}$ and $j_{l}\neq j_{k}\;\mathrm{mod}\;\frac{d}{m}$ for l≠k. Then, Rank(M)=min{s,t}.*

The proof of Lemma 3 is given in Appendix A. Since *F* is symmetric, a similar property can be obtained if one interchanges indices of the rows with that of columns of *M* in Equation (8). Lemma 3 means that *M* in Equation (8) is a row full-rank matrix or a column full-rank matrix.

## 3. Uncertainty Diagram of DFT

De Bièvre [20] has shown that the points on the hyperbola $n_{A}\left(\psi \right)n_{B}\left(\psi \right)=d$ belong to UNCD(A,B) of the DFT matrix *F*. He [24] also showed that A and B are completely incompatible if and only if

$$\begin{array}{c} \mathrm{UNCD}\left(A,B\right)=\{\left(n_{A},n_{B}\right)|n_{A}+n_{B}\geq d+1,n_{A},n_{B}\in Z_{d+1}^{*}\}. \end{array}$$

However, it is unclear if A and B are not completely incompatible. In this section, we continue to explore UNCD(A,B) of *F*.

The UNCD(A,B) of *F* is symmetric, since *F* is symmetric [24]. That is, $(n_{A},n_{B})\in$ UNCD(A,B) of *F* if and only if $(n_{B},n_{A})\in$ UNCD(A,B) of *F*.

**Theorem** **1.** *Suppose m|d and n≠0. A point $\left(d-n,n_{B}\right)$ belongs to the UNCD(A,B) of F if m|n and $\frac{n}{m}<n_{B}\leq \frac{d}{m}$.*

**Proof.**  In order to show $(d-n,n_{B})\in$ UNCD(A,B), we only need to find an $n\times n_{B}$ submatrix that satisfies Lemma 2. First of all, let

$$\begin{array}{cc} N= & F\left(\begin{array}{c} 0,1,\cdots ,\frac{n}{m}-1;\\ 0,m,\cdots ,(n_{B}-1)m. \end{array}\right)={\left(\begin{array}{c} \omega_{d}^{ikm} \end{array}\right)}_{\frac{n}{m}\times n_{B}}, \end{array}$$

where $i\in Z_{\frac{n}{m}}$ and $k\in Z_{n_{B}}$. Obviously, *N* is a $\frac{n}{m}\times n_{B}$ submatrix of *F*, since $\frac{n}{m}<n_{B}$, *N* is a Vandermonde Matrix that is a row full-rank matrix by Lemma 3. The matrix *N* has the following two properties:

(i) If row $i_{1}\in \left\{\frac{n}{m},\frac{n}{m}+1,\ldots ,\frac{d}{m}-1\right\}$ of *F* is added to *N* to obtain submatrix $N^{\prime }$, then Rank($N^{\prime }$) = Rank(*N*) + 1 = $\frac{n}{m}+1$ by Lemma 3. It is because $N^{\prime }$ is still a Vandermonde matrix, and $\frac{n}{m}+1\leq n_{B}$ and $\omega_{d}^{im}\neq \omega_{d}^{i^{\prime }m}$, where $i^{\prime },i\in Z_{\frac{n}{m}}⋃\left\{i_{1}\right\}$, $i^{\prime }\neq i$.

(ii) If a column of *N* is removed to obtain submatrix $N^{\prime \prime }$, then Rank($N^{\prime \prime }$) = Rank(*N*)$\;=\frac{n}{m}$ by Lemma 3 and $\frac{n}{m}\leq n_{B}-1$.

Secondly, consider the following $n\times n_{B}$ submatirx

(9) $$\begin{array}{ccc} M & = & F\left(\begin{array}{c} \cdots ,\frac{d}{m}i,\frac{d}{m}i+1,\cdots ,\frac{d}{m}i+\frac{n}{m}-1,\cdots ;\\ 0,m,\cdots ,(n_{B}-1)m. \end{array}\right),i=0,1,\ldots ,m-1\\ & = & \left(\begin{array}{c} N\\ N\\ \vdots \\ N \end{array}\right). \end{array}$$

The equality in Equation (9) holds due to $\omega_{d}^{(\frac{d}{m}i+j)\times km}=\omega_{d}^{j\times km}$. It follows Rank(*M*) = Rank(*N*) = $\frac{n}{m}<n_{B}$.

Since *N* has the above two properties and *M* has the form in Equation (9), we have Rank$\left(M^{\prime }\right)=\frac{n}{m}+1=$ Rank(M)+1 and Rank$\left(M^{\prime \prime }\right)=\frac{n}{m}=\;$Rank(M), where $M^{\prime }$ is obtained by adding a new row $\frac{d}{m}j+i_{1}$ to *M*, $i_{1}\in Z_{\frac{d}{m}}\backslash Z_{\frac{n}{m}}$, and $M^{\prime \prime }$ is obtained by removing a column of *M*. Here, we employ the condition $\omega_{d}^{(\frac{d}{m}j+i_{1})km}=\omega_{d}^{i_{1}km}$. So, *M* is the required submatrix. By Lemma 2, the required result is obtained. □

Notice that n≠0. This means Theorem 1 cannot work for $\left(d,n_{B}\right)$. However, taking m=1 and $n_{B}=d$ in Theorem 1, we obtain (i,d)∈ UNCD(A,B), where $i\in Z_{d}^{*}$. By the symmetry of UNCD(A,B) of *F*, we have (d,i)∈ UNCD(A,B), where $i\in Z_{d}^{*}$. In addition, (d,d)∈ UNCD(A,B) by Lemma 1.

Taking m=1 and $n_{A}=d-n$, we have the following result by Theorem 1 and the discussions above.

**Corollary** **1.** *A point $(n_{A},n_{B})\in$ UNCD(A,B) of F if $n_{A}+n_{B}\geq d+1$.*

**Figure 1:**
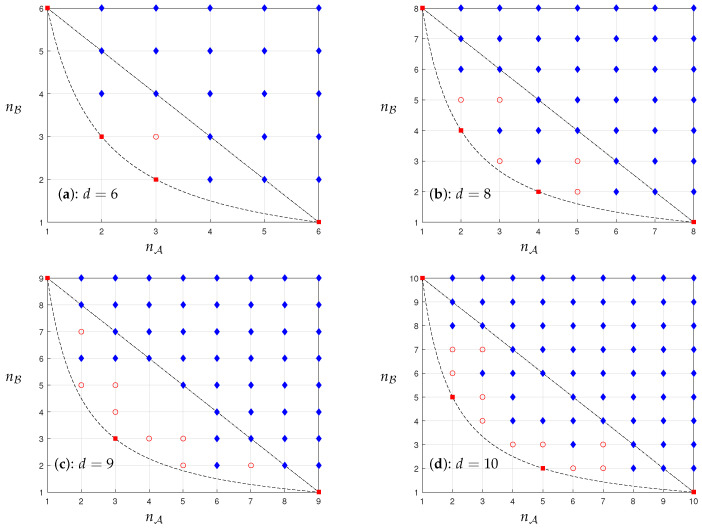
Uncertainty diagrams of the DFT matrix: (**a**) d=6; (**b**) d=8; (**c**) d=9; (**d**) d=10. Dashed curve is the hyperbola $n_{A}\left(\psi \right)n_{B}\left(\psi \right)=d$. Dot-dashed line is the line $n_{A}+n_{B}=d+1$. Red squares represent KD classical states. Blue diamonds represent KD nonclassical states. Red circles represent holes. The points without a mark enclosed by the hyperbola and the line on panel (**c**) and panel (**d**) imply that the existence of these points in UNCD(A,B) is unclear.

Note that in Corollary 1, $n_{A}$ can run over set $Z_{d+1}^{*}$ due to the above discussion of Corollary 1. Corollary 1 means that all the points above and on the line segment $n_{A}+n_{B}=d+1$ do exist, whether A and B are completely incompatible or not. It implies that there is no “hole” in the region of the $\left(n_{A},n_{B}\right)$ plane above and on the line $n_{A}+n_{B}=d+1$ for any *d*; that is, there is no absence of states with $\left(n_{A}\left(\psi \right),n_{B}\left(\psi \right)\right)$ in the region. The absence of states lies strictly above the hyperbola of $n_{A}\left(\psi \right)n_{B}\left(\psi \right)=d$ and strictly below the line $n_{A}+n_{B}=d+1$. This is illustrated in Figure 1. The following theorems show where the holes are.

**Theorem** **2.** *A point (d−n,2) belongs to the UNCD(A,B) of F if and only if n=0 or n|d but n≠d.*

**Proof.**  Sufficiency can be obtained by taking n=m in Theorem 1 and by the discussion above of Corollary 1 for n=0. We now show the necessity. Since (d,2) and (d−1,2) belong to UNCD(A,B), we have n=0,1, respectively. Then, we consider n≥2. A point (d−n,2) belongs to UNCD(A,B) of *F*. It means there exists an n×2 submatrix

$$\begin{array}{cc} M= & \left(\begin{array}{cc} \omega_{d}^{i_{0}j} & \omega_{d}^{i_{0}k}\\ \cdots & \cdots \\ \omega_{d}^{i_{n-1}j} & \omega_{d}^{i_{n-1}k} \end{array}\right). \end{array}$$

satisfying Lemma 2. Then, we have Rank(M)=1. This means that $\omega_{d}^{(i_{t}-i_{s})j}=\omega_{d}^{(i_{t}-i_{s})k}$ for any $s,t\in Z_{n}$ and s≠t. It follows that $\left(i_{t}-i_{s}\right)\left(k-j\right)\equiv 0\;\mathrm{mod}\;d$. Assume gcd(k−j,d)=p. Then, $i_{s}\equiv i_{t}\;\mathrm{mod}\;\frac{d}{p}$. That is, $i_{s}$ is in the congruence class of $i_{t}$ modulo $\frac{d}{p}$. Notice that the cardinality of the congruence class of $i_{t}$ modulo $\frac{d}{p}$ is *p*. It implies that there are at most *p* rows in submatrix *M* by the arbitrariness of $s,t\in Z_{n}$, i.e., n≤p. In fact, the submatrix *M* has only *p* rows, i.e., n=p. Otherwise, *M* cannot satisfy the second condition of Lemma 2. Thus, n|d since p|d. However, n=p≠d, since j≠kmodd. □

Note that for the UNCD(A,B) of *F*, $n_{A}\left(\psi \right)n_{B}\left(\psi \right)\geq d$ for any state |ψ〉∈H. Thus, n=d is meaningless in Theorem 2. This means that a point (d−n,2)∉ the UNCD(A,B) of *F* if and only if n∤d. In fact, we only consider the case $n\leq \frac{d}{2}$ since (d−n)×2≥d. For example, if d=6, there is no hole for $n_{B}=2$, since n=1,2,3 are all the divisors of 6. See panel (a) of Figure 1. If d=8, there is a hole (5,2), since n=3∤8. See panel (b) of Figure 1.

**Theorem** **3.** *Suppose d has only nontrivial prime divisors. Then, a point (d−n,3) belongs to the UNCD(A,B) of F if and only if n=0 or there exists a divisor m of d such that 3m≤d and n=m or n=2m.*

The proof of Theorem 3 is given in Appendix B. Theorem 3 presents where the holes are when $n_{B}=3$. For instance, if d=9, m=1,2. Then, n=0,1,2,3,6. It implies that $n_{A}=9,8,7,6,3$, but $n_{A}\neq 5,4$. Thus, there is two holes, (5,3) and (4,3), for $n_{B}=3$. See panel (c) of Figure 1. Panel (d) is for d=10. It should be noted that although 8 has a nontrivial nonprime divisor, one can check that the result of Theorem 3 for d=8 still holds, since the proof can be followed similarly for the proof of Theorem 3.

## 4. KD Nonclassicality on DFT

In Section 3, the existence of states in UNCD(A,B) has been shown. In this section, we focus on the KD nonclassicality of a state based on DFT. In Ref. [20], De Bièvre gave a conjecture, that is, whether it is true that the only KD classical states for the DFT are the ones on the hyperbola $n_{A}\left(\psi \right)n_{B}\left(\psi \right)=d$. From a different perspective than Xu [25], we give an alternative method to prove this conjecture. Our method avoids analyzing the phases and only uses the concept of congruence class.

**Theorem** **4.** *Suppose that the bases A and B are related by the DFT matrix F. A state |ψ〉∈H is KD nonclassical if and only if $n_{A}\left(\psi \right)n_{B}\left(\psi \right)>d$. In other words, |ψ〉 is KD classical if and only if $n_{A}\left(\psi \right)n_{B}\left(\psi \right)=d$.*

**Proof.**  The necessity has been proved by De Bièvre in Ref. [20]. Here, we only need to show the sufficiency, i.e., |ψ〉∈H is KD nonclassical if $n_{A}\left(\psi \right)n_{B}\left(\psi \right)>d$.

We proceed by contradiction. Suppose that |ψ〉 is KD classical, i.e.,

$$\langle a_{i}|\psi \rangle \langle \psi |b_{j}\rangle \langle b_{j}|a_{i}\rangle \geq 0$$

for any $i,j\in Z_{d}$. Since the KD distribution is insensitive to global phase rotations, we perform global phase rotations $|a_{i}\rangle \to e^{\sqrt{-1}\phi_{i}}|a_{i}\rangle$ and $|b_{j}\rangle |\to e^{\sqrt{-1}\phi_{j}}|b_{j}\rangle |$ such that $\langle a_{i}|\psi \rangle$ and $\langle \psi |b_{j}\rangle$ are non-negative for $i,j\in Z_{d}$. Reordering the basis vectors, we can suppose that $\langle a_{i_{m}}|\psi \rangle >0$ and $\langle \psi |b_{j_{s}}\rangle >0$ for $m\in Z_{n_{A}\left(\psi \right)}$ and $s\in Z_{n_{B}\left(\psi \right)}$, where $i_{m},j_{s}$ are initial indices of basis vectors $|a_{m}\rangle$ and $|b_{s}\rangle$, respectively. Thus, for the same range of $i_{m}$ and $j_{s}$, we have $\langle b_{j_{s}}|a_{i_{m}}\rangle \geq 0$. Since *F* is the DFT matrix, we have $|F_{ij}\left|=\right|\langle a_{i}|b_{j}\rangle |=\frac{1}{\sqrt{d}}\left|\omega_{d}^{ij}\right|=\frac{1}{\sqrt{d}}$. It follows $\langle a_{i_{m}}|b_{j_{s}}\rangle =\frac{1}{\sqrt{d}}$ for $m\in Z_{n_{A}\left(\psi \right)}$, and $s\in Z_{n_{B}\left(\psi \right)}$. It means that the top left-hand block $V=(v_{ij})$ in the new transition matrix after reordering the basis vectors is an $n_{A}\times n_{B}$ submatrix with all entries of $\frac{1}{\sqrt{d}}$.

Let us first consider the trivial case. If $n_{A}\left(\psi \right)=1$ (or $n_{B}\left(\psi \right)=1$), then $n_{B}\left(\psi \right)=d$ (or $n_{A}\left(\psi \right)=d$), since *F* is the DFT matrix. Thus, $n_{A}\left(\psi \right)n_{B}\left(\psi \right)=d$. It is a contradiction with $n_{A}\left(\psi \right)n_{B}\left(\psi \right)>d$.

Next, we consider the case $n_{A}\left(\psi \right)\geq 2$ and $n_{B}\left(\psi \right)\geq 2$. For any $m\in Z_{n_{A}\left(\psi \right)}$, any $s,t\in Z_{n_{B}\left(\psi \right)}$ with s<t, calculate the product of two numbers, $\sqrt{d}v_{ms}^{*}$ and $\sqrt{d}v_{mt}$, in *V*. We have

(10) $$\begin{array}{cc} 1=\; & dv_{ms}^{*}\cdot v_{mt}\\ =\; & de^{-\sqrt{-1}\left(\phi_{j_{s}}-\phi_{i_{m}}\right)}\langle b_{j_{s}}|a_{i_{m}}\rangle e^{\sqrt{-1}\left(\phi_{j_{t}}-\phi_{i_{m}}\right)}\langle a_{i_{m}}|b_{j_{t}}\rangle \\ =\; & e^{\sqrt{-1}\left(\phi_{j_{t}}-\phi_{j_{s}}\right)}\omega_{d}^{-i_{m}j_{s}}\omega_{d}^{i_{m}j_{t}}\\ =\; & \omega_{d}^{\alpha_{s,t}+i_{m}\left(j_{t}-j_{s}\right)}. \end{array}$$

where $\alpha_{s,t}:=\frac{d}{2\pi }\left(\phi_{j_{t}}-\phi_{j_{s}}\right)$. This implies that $\alpha_{s,t}+i_{m}\left(j_{t}-j_{s}\right)\equiv 0\;\mathrm{mod}\;d$. Notice that $\alpha_{s,t}$ is independent of *m*. Thus, for any $m,n\in Z_{n_{A}\left(\psi \right)}$ and m<n, we have

(11) $$\begin{array}{ccc} \alpha_{s,t}+i_{m}\left(j_{t}-j_{s}\right) & \equiv & 0\;\mathrm{mod}\;d,\\ \alpha_{s,t}+i_{n}\left(j_{t}-j_{s}\right) & \equiv & 0\;\mathrm{mod}\;d. \end{array}$$

It follows that

(12) $$\left(i_{n}-i_{m}\right)\left(j_{t}-j_{s}\right)\equiv 0\;\mathrm{mod}\;d.$$

Suppose gcd$(j_{t}-j_{s},d)=p$ and $q:=\frac{d}{p}$. If p=1, then $i_{n}\equiv i_{m}\;\mathrm{mod}\;d$. This is impossible, since $m,n\in Z_{n_{A}\left(\psi \right)}$ and m<n. If p≠1, then we have

(13) $$\begin{array}{ccc} i_{n} & \equiv & i_{m}\;\mathrm{mod}\;q,\\ j_{t} & \equiv & j_{s}\;\mathrm{mod}\;p. \end{array}$$

It implies that $i_{n}$ is in the congruence class of $i_{m}$ modulo *q* and the cardinality of the congruence class of $i_{m}$ is *p*. Similarly, $j_{t}$ is in the congruence class of $j_{s}$ modulo *p*, and the cardinality of the congruence class of $j_{s}$ is *q*. Because of the arbitrariness of $i_{n},i_{m}\in Z_{n_{A}\left(\psi \right)}$ and $j_{t},j_{s}\in Z_{n_{B}\left(\psi \right)}$, we obtain $n_{A}\left(\psi \right)\leq p$ and $n_{B}\left(\psi \right)\leq q$. Therefore, $n_{A}\left(\psi \right)n_{B}\left(\psi \right)\leq d$. This is a contradiction with $n_{A}\left(\psi \right)n_{B}\left(\psi \right)>d$. □

From the above proof, we find that $n_{A}\left(\psi \right)n_{B}\left(\psi \right)\leq d$ if |ψ〉 is KD classical. It follows that $n_{A}\left(\psi \right)n_{B}\left(\psi \right)=d$, since $n_{A}\left(\psi \right)n_{B}\left(\psi \right)\geq d$ for the DFT matrix. This implies that only the KD classical states lie on the hyperbola of $n_{A}\left(\psi \right)n_{B}\left(\psi \right)=d$. This result gives a positive answer to the conjecture in Ref. [20].

When *d* is prime, the bases A,B are completely incompatible. Then, all the states are nonclassical except for basis vectors [20]. In Theorem 4, the KD nonclassicality of a state based on the DFT matrix, whenever *d* is prime or not, is completely characterized by Donoho and Stark’s product uncertainty relation [27], $n_{A}n_{B}=d$. See Figure 1. It should be noted that this method is completely different from the method in Ref. [25]. Here, we only use the concept of congruence class.

In the following example, we analyze the positions of two states in UNCD(A,B) and their KD nonclassicality.

**Example** **1.** *Consider the computational basis ${A=\{|j\rangle \}}_{j=0}^{5}$ and $B=\{|\Phi_{j}\rangle {\}}_{j=0}^{5}$ in $C^{6}$, where $|\Phi_{j}\rangle =\frac{1}{\sqrt{6}}\Sigma_{k=0}^{5}\omega_{6}^{jk}|k\rangle$. Obviously, the DFT matrix F is the transition matrix from A to B. Take a state $|\psi_{1}\rangle =\frac{1}{\sqrt{2}}[|\Phi_{0}\rangle -|\Phi_{2}\rangle ]=\frac{1}{\sqrt{12}}[\left(1-\omega_{6}^{2}\right)|1\rangle +\left(1+\omega_{6}\right)|2\rangle +\left(1-\omega_{6}^{2}\right)|4\rangle +\left(1+\omega_{6}\right)\left|5\rangle \right]$. Then, $n_{A}\left(\psi_{1}\right)=4$ and $n_{B}\left(\psi_{1}\right)=2$. The KD distribution, presented in Table 1, is nonclassical. Take $|\psi_{2}\rangle =\frac{1}{\sqrt{2}}[|\Phi_{0}\rangle +|\Phi_{3}\rangle ]=\frac{1}{\sqrt{3}}[|0\rangle +|2\rangle +\left|4\rangle \right]$, then $n_{A}\left(\psi_{2}\right)=3$ and $n_{B}\left(\psi_{2}\right)=2$. The KD distribution, presented in Table 2, is real and non-negative.*

It should be noted that the notions of nonclassicality based on the different contexts are different. In Ref. [30], Ferraro et al. focus on two celebrated criteria for defining the nonclassicality of *bipartite* bosonic quantum systems. One stems from physical constraints on the quantum phase space. The other stems from information theoretic concepts. They showed that the two defining criteria are maximally inequivalent. Notice that these two types of definitions of the nonclassicality are for bipartite quantum systems. The quasiprobability formalism provides a useful alternative to describe the nonclassicality of quantum states. The Wigner function is the most famous quasiprobability distribution which deals with continuous variable systems [3,4]. However, it is ill-suited for finite-dimensional systems and observables. The KD distribution can deal with finite-dimensional systems. It is a versatile tool in studying quantum information processing. Recently, Budiyono et al. quantified quantum coherence via KD quasiprobability [31]. Since the terms “classicality” and “nonclassicality” lack unique definition, Langrenez et al. changed the terminology from KD-classical to KD-positive [26]. Notice that the KD distribution can be used for a single qudit system. It implies that a finite-dimensional system is not always nonclassical. For instance, the KD classical states only lie on the hyperbola of $n_{A}\left(\psi \right)n_{B}\left(\psi \right)=d$ for the DFT matrix. See Figure 1.

## 5. Conclusions and Discussion

We studied the uncertainty diagram and Kirkwood–Dirac nonclassicality based on DFT in a *d*-dimensional system. We showed that for the uncertainty diagram of the DFT matrix, there is no “hole” in the region of the $\left(n_{A},n_{B}\right)$ plane above and on the line $n_{A}+n_{B}=d+1$, whether the bases A,B are completely incompatible bases or not. The absence of states lies strictly above the hyperbola of $n_{A}\left(\psi \right)n_{B}\left(\psi \right)=d$ and strictly below the line $n_{A}+n_{B}=d+1$. Then, we showed where the holes are when $n_{B}=2,3$. Finally, an alternative method to prove the conjecture in Ref. [20] was proposed.

As is known, completely incompatible bases imply that repeated compatible selective measurements cannot occur. However, for the DFT matrix with nonprime *d*, A and B are not completely incompatible. This means that there exist some states such that repeated compatible selective measurements can occur. In addition, as concerns the KD distribution, nonclassical KD quasiprobabilities have been linked to various forms of quantum advantages in weak measurements [15,16,17], quantum tomography [11,12] and quantum metrology [32]. Our results might provide better understanding and insight into the roles of KD nonclassicality as a resource in quantum information processing. We hope our results can lead to more findings in this field. There are still some questions left. For example, how is the strength of the KD nonclassicality established?

## Figures and Tables

**Table 1:** The KD distribution of $|\psi_{1}\rangle =\frac{1}{\sqrt{2}}[|\Phi_{0}\rangle -|\Phi_{2}\rangle ]$.

$Q_{\mathrm{ij}}\left(\psi_{1}\right)$	|0〉	|1〉	|2〉	|3〉	|4〉	|5〉
$|\Phi_{0}\rangle$	0	$\frac{1}{12}\left(1-\omega_{6}^{2}\right)$	$\frac{1}{12}\left(1+\omega_{6}\right)$	0	$\frac{1}{12}\left(1-\omega_{6}^{2}\right)$	$\frac{1}{12}\left(1+\omega_{6}\right)$
$|\Phi_{1}\rangle$	0	0	0	0	0	0
$|\Phi_{2}\rangle$	0	$\frac{1}{12}\left(1+\omega_{6}\right)$	$\frac{1}{12}\left(1-\omega_{6}^{2}\right)$	0	$\frac{1}{12}\left(1+\omega_{6}\right)$	$\frac{1}{12}\left(1-\omega_{6}^{2}\right)$
$|\Phi_{3}\rangle$	0	0	0	0	0	0
$|\Phi_{4}\rangle$	0	0	0	0	0	0
$|\Phi_{5}\rangle$	0	0	0	0	0	0

**Table 2:** The KD distribution of $|\psi_{2}\rangle =\frac{1}{\sqrt{2}}[|\Phi_{0}\rangle +|\Phi_{3}\rangle ]$.

$Q_{\mathrm{ij}}\left(\psi_{2}\right)$	|0〉	|1〉	|2〉	|3〉	|4〉	|5〉
$|\Phi_{0}\rangle$	$\frac{1}{6}$	0	$\frac{1}{6}$	0	$\frac{1}{6}$	0
$|\Phi_{1}\rangle$	0	0	0	0	0	0
$|\Phi_{2}\rangle$	0	0	0	0	0	0
$|\Phi_{3}\rangle$	$\frac{1}{6}$	0	$\frac{1}{6}$	0	$\frac{1}{6}$	0
$|\Phi_{4}\rangle$	0	0	0	0	0	0
$|\Phi_{5}\rangle$	0	0	0	0	0	0

## Data Availability

The datasets generated during and/or analyzed during the current study are available from the corresponding author on reasonable request.

## Appendix A. The Proof of Lemma 3

**Proof** **of** **Lemma** **3.** Suppose min{s,t}=u. Consider the u×u submatrix $\tilde{M}$ in Equation (8),

$$\begin{array}{cc} \tilde{M}= & F\left(\begin{array}{c} i_{0},i_{0}+m,\cdots ,i_{0}+\left(u-1\right)m;\\ j_{0},j_{1},\cdots ,j_{u-1}. \end{array}\right)\\ = & \left(\begin{array}{ccc} \omega_{d}^{i_{0}j_{0}} & \cdots & \omega_{d}^{i_{0}j_{u-1}}\\ \cdots & \cdots & \cdots \\ \;\omega_{d}^{i_{0}j_{0}+\left(u-1\right)mj_{0}} & \cdots & \omega_{d}^{i_{0}j_{u-1}+\left(u-1\right)mj_{u-1}} \end{array}\right). \end{array}$$

Notice that

$$\begin{array}{ccc} \det(\tilde{M}) & = & \omega_{d}^{i_{0}\left(j_{0}+\ldots +j_{u-1}\right)}\prod_{0\leq l<k\leq u-1}\left(\omega_{d}^{mj_{k}}-\omega_{d}^{mj_{l}}\right)\\ & = & \omega_{d}^{i_{0}\left(j_{0}+\ldots +j_{u-1}\right)}\prod_{0\leq l<k\leq u-1}\left(\omega_{\frac{d}{m}}^{j_{k}}-\omega_{\frac{d}{m}}^{j_{l}}\right)\neq 0 \end{array}$$

since $\omega_{\frac{d}{m}}^{j_{k}}\neq \omega_{\frac{d}{m}}^{j_{l}}\;\mathrm{mod}\;\frac{d}{m}$. Thus Rank($M^{\prime }$) = *u*. The desired result is obtained. □

## Appendix B. The Proof of Theorem 3

**Proof** **of** **Theorem** **3.** Sufficiency can be obtained by Theorem 1 and the discussion above on Corollary 1. Now, we consider the necessity. Since (d,3) belongs to UNCD(A,B), we have n=0. If n=1 or 2, we can take m=1 such that the result holds. For n≥3, a point (d−n,3) belongs to the UNCD(A,B) of *F*. This means there exists an n×3 submatrix

$$\begin{array}{cc} M= & \left(\begin{array}{ccc} \omega_{d}^{i_{0}j_{0}} & \omega_{d}^{i_{0}j_{1}} & \omega_{d}^{i_{0}j_{2}}\\ \cdots & \cdots & \cdots \\ \omega_{d}^{i_{n-1}j_{0}} & \omega_{d}^{i_{n-1}j_{1}} & \omega_{d}^{i_{n-1}j_{2}} \end{array}\right). \end{array}$$

satisfying Lemma 2. Then, Rank(M)≤2. Without loss of generality, assume that the first column of *M* is a linear combination of the other two columns. It follows that $\omega_{d}^{i_{k}j_{0}}=c_{1}\omega_{d}^{i_{k}j_{1}}+c_{2}\omega_{d}^{i_{k}j_{2}}$, where k=0,…,n−1, and $c_{1},c_{2}$ are the coefficients of the linear combination. Then, $c_{1}\omega_{d}^{i_{k}\left(j_{1}-j_{0}\right)}+c_{2}\omega_{d}^{i_{k}\left(j_{2}-j_{0}\right)}=1$. By Euler’s formula $e^{x\sqrt{-1}}=\cos x$+$\sqrt{-1}\sin x$, we obtain

(A1) $$\begin{array}{ccc} c_{1}\cos\frac{2\pi i_{k}\left(j_{1}-j_{0}\right)}{d}+c_{2}\cos\frac{2\pi i_{k}\left(j_{2}-j_{0}\right)}{d} & = & 1,\\ c_{1}\sin\frac{2\pi i_{k}\left(j_{1}-j_{0}\right)}{d}+c_{2}\sin\frac{2\pi i_{k}\left(j_{2}-j_{0}\right)}{d} & = & 0. \end{array}$$

Calculating the square of two sides of the two equations in Equation (A1) and then adding the two equations, we obtain

(A2) $$\begin{array}{c} 2c_{1}c_{2}\cos\frac{2\pi i_{k}\left(j_{1}-j_{2}\right)}{d}=1-c_{1}^{2}-c_{2}^{2}, \end{array}$$

Case 1. If $c_{1}c_{2}=0$, then $c_{1}=0$ or $c_{2}=0$. Without loss of generality, suppose $c_{1}=0$. Then, $\omega^{i_{k}\left(j_{0}-j_{2}\right)}=c_{2}$. For $i_{k}\neq i_{l}$, $\omega^{\left(i_{k}-i_{l}\right)\left(j_{0}-j_{2}\right)}=1$. It follows that $\left(i_{k}-i_{l}\right)\left(j_{0}-j_{2}\right)\equiv 0\;\mathrm{mod}\;d$ for any $k,l\in Z_{n}$. Let $\gcd(j_{2}-j_{0},d)=p$. We have $j_{2}\equiv j_{0}\;\mathrm{mod}\;p$ and $i_{k}\equiv i_{l}\;\mathrm{mod}\;\frac{d}{p}$. This means that $i_{k}$ is in the congruence class of $i_{l}$ modulo $\frac{d}{p}$ for any $k,l\in Z_{n}$. This implies that n≤p, since the cardinality of the congruence class $i_{l}$ modulo $\frac{d}{p}$ is *p*. Next, we show that it is impossible for n<p. Since $c_{1}=0$, Rank(M) must be 1. Otherwise, if Rank(M)=2, we can remove column $j_{1}$, and then Rank$(M^{\prime \prime })=1$, where $M^{\prime \prime }$ is a new submatrix of *F* that is obtained by removing column $j_{1}$ of *M*. It contradicts condition (iii) of Lemma 2. For any $k,l\in Z_{n}$, the row $i_{k}$ of *M* is proportional to row $i_{l}$, since Rank(M)=1. That is, $\omega_{d}^{\left(i_{k}-i_{l}\right)j_{0}}=\omega_{d}^{\left(i_{k}-i_{l}\right)j_{1}}=\omega_{d}^{\left(i_{k}-i_{l}\right)j_{2}}$. Hence, $\left(i_{k}-i_{l}\right)\left(j_{0}-j_{1}\right)\equiv 0\;\mathrm{mod}\;d$. It follows that $j_{0}\equiv j_{1}\equiv j_{2}\;\mathrm{mod}\;p$, since *p* is prime. If n<p, it means that there exists at least one row, say row $i_{s}$, that is not in submatrix *M* but satisfies $i_{s}\equiv i_{k}\;\mathrm{mod}\;\frac{d}{p}$. Thus, row $i_{s}$ can be added to *M* to obtain submatrix $M^{\prime }$ such that Rank$(M^{\prime })=$ Rank(M), since $\omega_{d}^{\left(i_{k}-i_{s}\right)j_{0}}=\omega_{d}^{\left(i_{k}-i_{s}\right)j_{1}}=\omega_{d}^{\left(i_{k}-i_{s}\right)j_{2}}$. This contradicts condition (ii) of Lemma 2. It implies n=p. The required divisor *m* of *d* is *p*, satisfying m=n and 3m≤d. Case 2. If $c_{1}c_{2}\neq 0$, from Equation (A2), we have

(A3) $$\begin{array}{c} \cos\frac{2\pi i_{k}\left(j_{1}-j_{2}\right)}{d}=\frac{1-c_{1}^{2}-c_{2}^{2}}{2c_{1}c_{2}}, \end{array}$$

It follows that

(A4) $$\begin{array}{c} \cos\frac{2\pi i_{k}\left(j_{1}-j_{2}\right)}{d}=\cos\frac{2\pi i_{l}\left(j_{1}-j_{2}\right)}{d},k,l\in Z_{n}. \end{array}$$

Thus, we have

(A5) $$\begin{array}{c} \left(i_{k}\pm i_{l}\right)\left(j_{1}-j_{2}\right)\equiv 0\;\mathrm{mod}\;d. \end{array}$$

Suppose $\gcd(j_{1}-j_{2},d)=p$. Then, $j_{2}\equiv j_{1}\;\mathrm{mod}\;p$ and $i_{k}\equiv \pm i_{l}\;\mathrm{mod}\;\frac{d}{p}$. By Equation (A5), there are two subcases, as follows. Case 2.1. If $i_{k}\equiv i_{l}\;\mathrm{mod}\;\frac{d}{p}$ for any $k,l\in Z_{n}$, by assumption, we have

$$\begin{array}{ccc} \omega_{d}^{i_{k}j_{0}} & = & c_{1}\omega_{d}^{i_{k}j_{1}}+c_{2}\omega_{d}^{i_{k}j_{2}}=\omega_{d}^{i_{k}j_{2}}\left(c_{1}\omega_{d}^{i_{k}\left(j_{1}-j_{2}\right)}+c_{2}\right)\\ \omega_{d}^{i_{l}j_{0}} & = & c_{1}\omega_{d}^{i_{l}j_{1}}+c_{2}\omega_{d}^{i_{l}j_{2}}=\omega_{d}^{i_{l}j_{2}}\left(c_{1}\omega_{d}^{i_{l}\left(j_{1}-j_{2}\right)}+c_{2}\right). \end{array}$$

Notice that $\omega_{d}^{i_{k}\left(j_{1}-j_{2}\right)}=\omega_{d}^{i_{l}\left(j_{1}-j_{2}\right)}$, since $j_{2}\equiv j_{1}\;\mathrm{mod}\;p$ and $i_{k}\equiv i_{l}\;\mathrm{mod}\;\frac{d}{p}$. It follows that $c_{1}\omega_{d}^{i_{k}\left(j_{1}-j_{2}\right)}+c_{2}=c_{1}\omega_{d}^{i_{l}\left(j_{1}-j_{2}\right)}+c_{2}$. Then, $\omega_{d}^{\left(i_{k}-i_{l}\right)j_{0}}=\omega_{d}^{\left(i_{k}-i_{l}\right)j_{2}}$. It follows that $j_{0}\equiv j_{1}\equiv j_{2}\;\mathrm{mod}\;p$, since *p* is prime. A similar discussion in Case 1 can be applied here. We can obtain n=p=m. Case 2.2. If this is not the case, there exists an index set $C\subseteq Z_{n}$ such that $i_{k}\equiv i_{0}\;\mathrm{mod}\;\frac{d}{p},k\in C$ and $i_{l}\equiv -i_{0}\;\mathrm{mod}\;\frac{d}{p},l\in Z_{n}\backslash C$. Applying a similar discussion in Case 2.1 for $i_{k}\equiv i_{0}\;\mathrm{mod}\;\frac{d}{p},k\in C$, we have that the cardinality of C is *p*. Similarly, the cardinality of $Z_{n}\backslash C$ is also *p*. Thus, n=2p. The required divisor *m* of *d* is *p*, satisfying 2m=n and 3m≤d. □
